# Supplementary material for: Machine Learning Method to Explore the Correlation between Fly Ash Content and Chloride Resistance
Source: Materials (Basel). 2024 Mar 4;17(5):1192. doi: 10.3390/ma17051192 (PMC10934373; doi:10.3390/ma17051192)
Supplement: Supplementary file 1 [file materials-17-01192-s001.zip › materials-2865984-supplementary.pdf]

Machine Learning Method to Explore the Correlation between Fly Ash Content and Chloride Resistance

Ruiqi Wang [1], Yupeng Huo [1], Teng Wang [1], Peng Hou [2], Zuo Gong [2], Guodong Li [1,*] and Changyan Li [2,*]

[1] College of Transportation, Inner Mongolia University, Hohhot 010031, China; 32015127@mail.imu.edu.cn (R.W.); 32124006@mail.imu.edu.cn (Y.H.); 32124046@mail.imu.edu.cn (T.W.)

[2] College of Chemistry and Chemical Engineering, Inner Mongolia University, Hohhot 010031, China; 32207133@mail.imu.edu.cn (P.H.); 32207131@mail.imu.edu.cn (Z.G.)

* Correspondence: lgd567@imu.edu.cn (G.L.); celicy@imu.edu.cn (C.L.)

$$R^2 = 1 - \frac{\sum_{i=1}^{n}\left(y_i' - y_i\right)^2}{\sum_{i=1}^{n}\left(y_i - \overline{y}\right)^2} \qquad \text{(Seq.S1)}$$

$$MSE = \frac{1}{n}\sum_{i=1}^{n}\left(y_i' - y_i\right)^2 \qquad \text{(Seq.S2)}$$

$$RMSE = \sqrt{\frac{1}{n}\sum_{i=1}^{n}\left(y_i' - y_i\right)^2} \qquad \text{(Seq.S3)}$$

$$MAE = \frac{1}{n}\sum_{i=1}^{n}\left|y_i' - y_i\right| \qquad \text{(Seq.S4)}$$

$$y = 0.00486 + \frac{0.19163}{0.24782}\mathrm{e}^{-2\left(\frac{x+0.00308}{0.24782}\right)^2} \qquad \text{(Seq.S5)}$$

$$y = 0.00618 + \frac{0.04571}{0.06251}\mathrm{e}^{-2\left(\frac{x-0.000359}{0.06251}\right)^2} \qquad \text{(Seq.S6)}$$

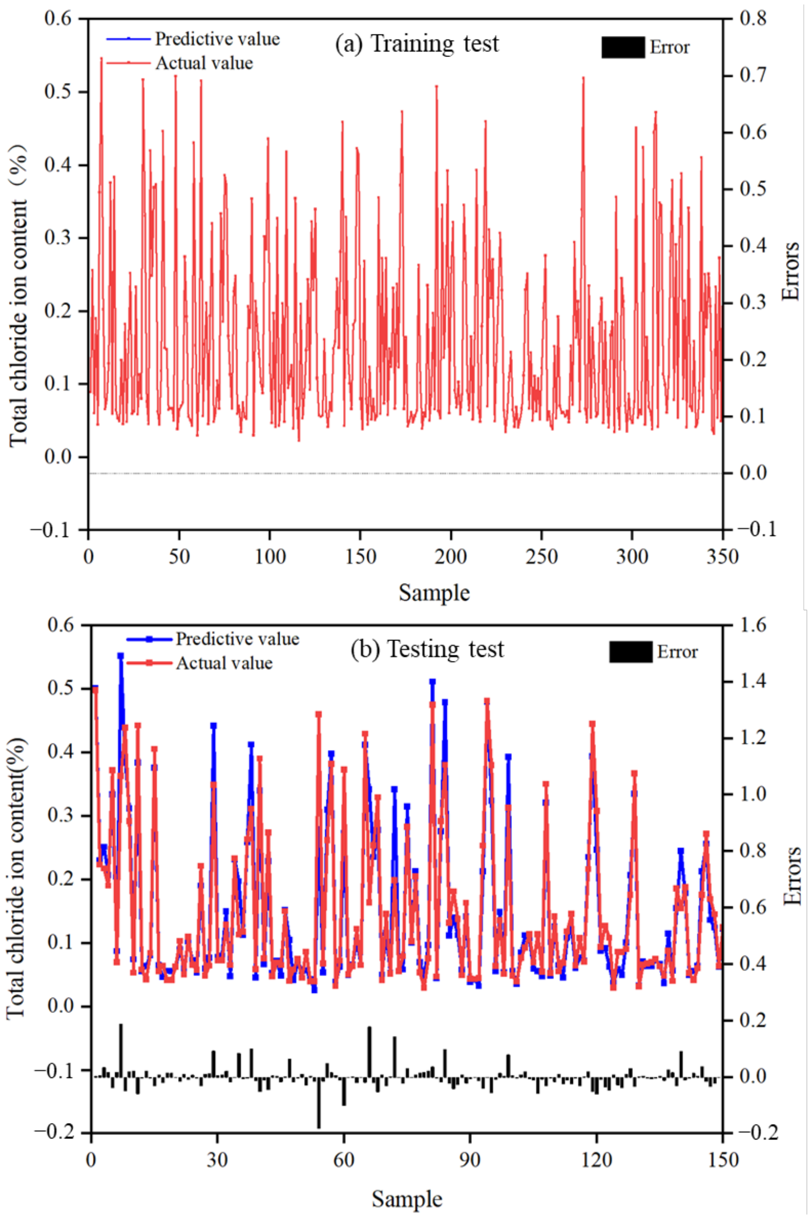

Figure S1. Target and predicted total chloride ion content calculated by GBR

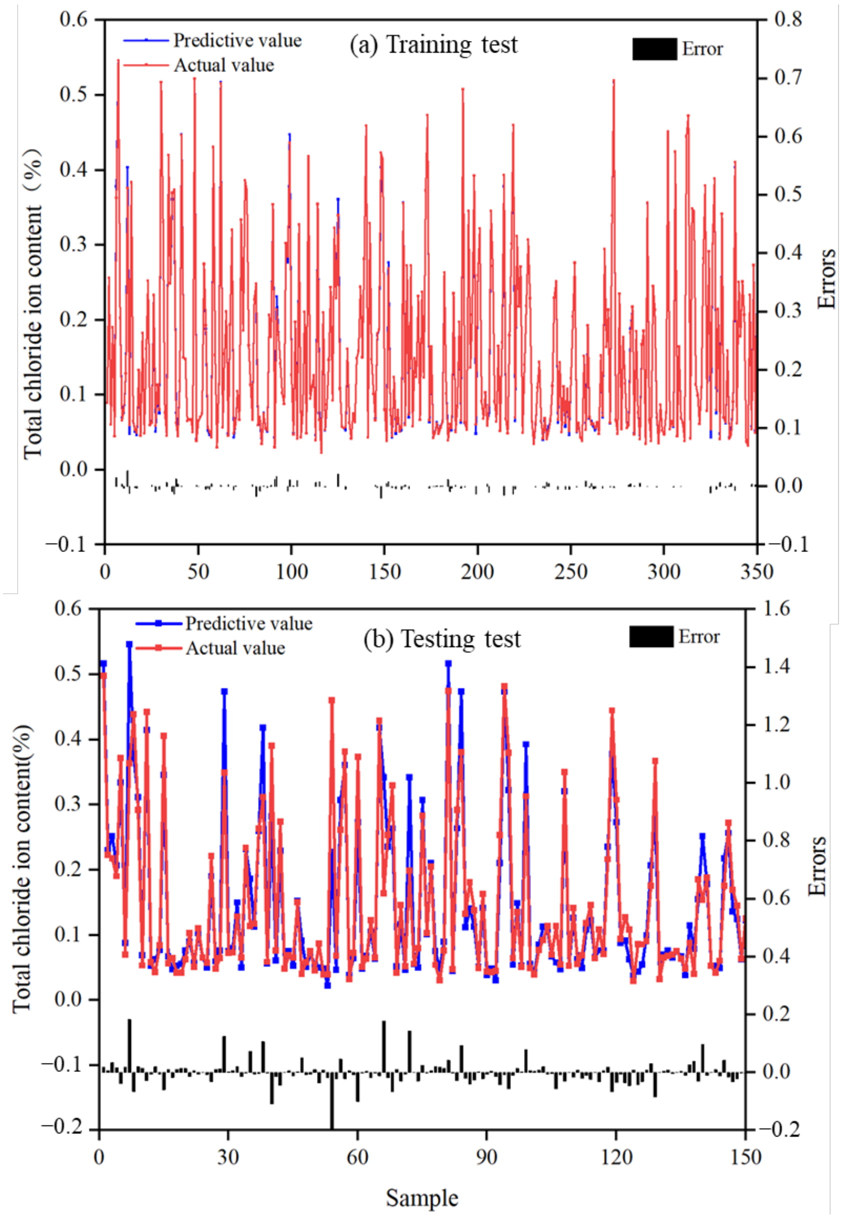

Figure S2. Target and predicted total chloride ion content calculated by DT

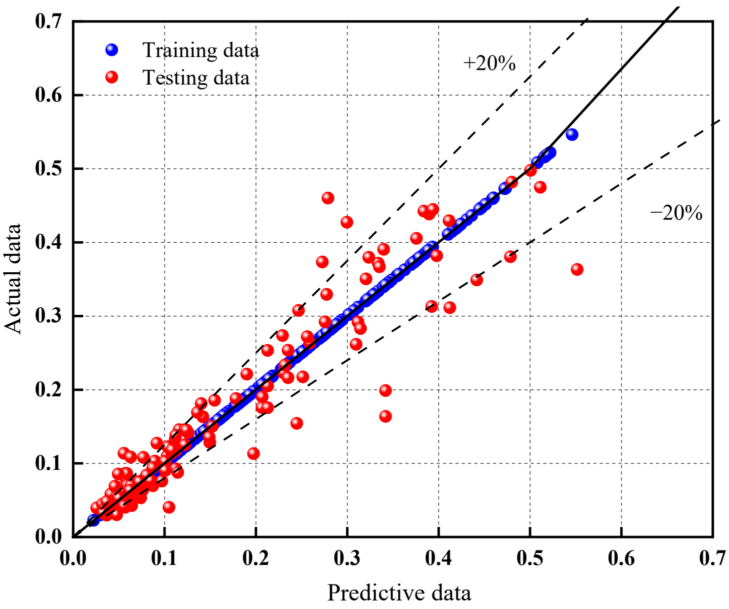

Figure S3. Measured and predicted values of GBR model

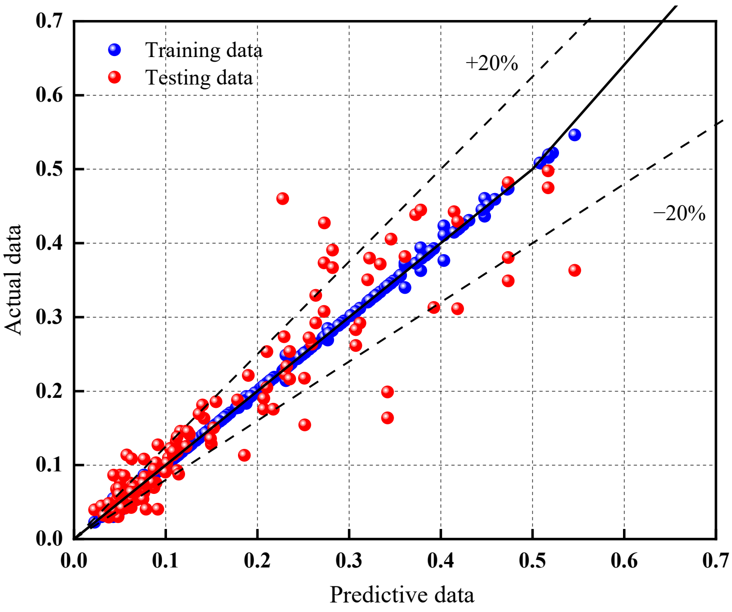

Figure S4. Measured and predicted values of DT model

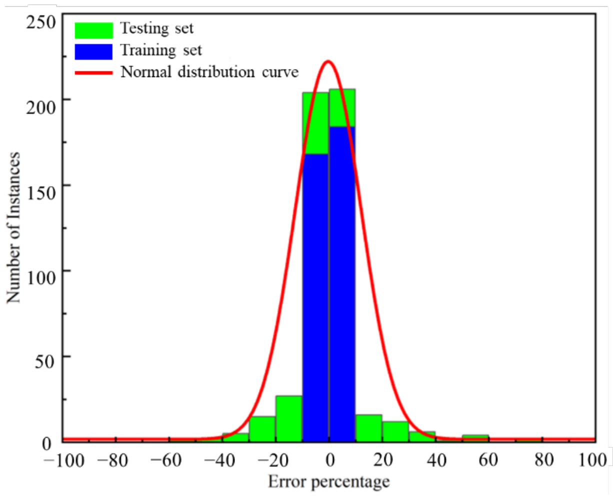

Figure S5. Error percentage distribution and normal distribution fitting of GBR model

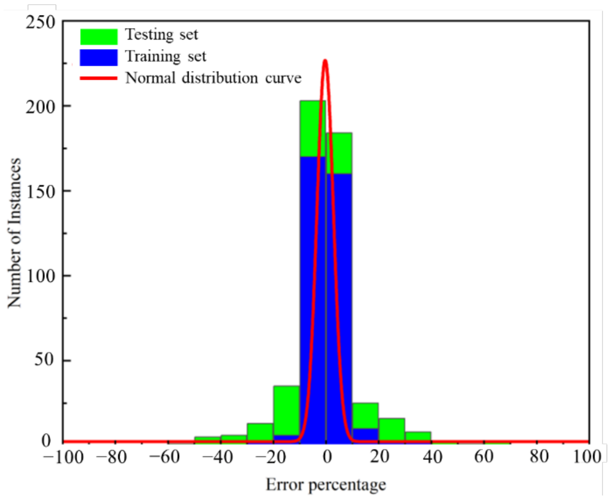

Figure S6. Error percentage distribution and normal distribution fitting of DT model

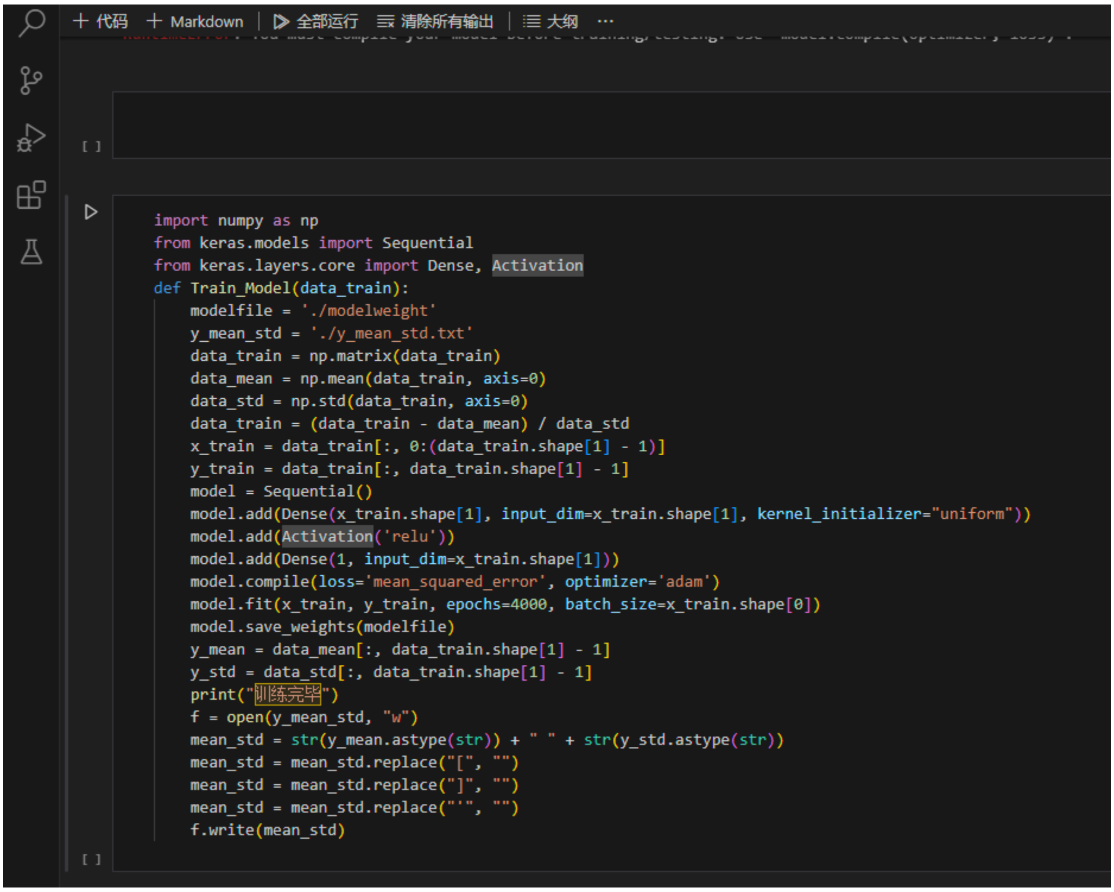

Figure S7. Python coding
